# Supplementary material for: Associations between Prediagnostic Circulating Bilirubin Levels and Risk of Gastrointestinal Cancers in the UK Biobank
Source: Cancers (Basel). 2021 Jun 1;13(11):2749. doi: 10.3390/cancers13112749 (PMC8198711; doi:10.3390/cancers13112749)
Supplement: Supplementary file 1 [file cancers-13-02749-s001.zip › cancers-1188921-supplementary.pdf]

Associations between prediagnostic circulating bilirubin levels and risk of gastrointestinal cancers in the UK Biobank

Nazlisadat Seyed Khoei[1], Karl-Heinz Wagner[1], Robert Carreras-Torres[2], Marc J. Gunter[3], Neil Murphy[3*], Heinz Freisling [3*]

Supplemental materials:

Tables S1. The association between circulation total bilirubin levels and gastrointestinal cancers risk across strata of potential effect modifiers in the UK Biobank

Table S2. Risk (hazard ratios) of gastrointestinal cancers associated with circulating bilirubin levels corrected for regression dilution in the UK Biobank

Table S3. Risk (hazard ratios) of gastrointestinal cancers associated with circulating direct bilirubin levels in the UK Biobank

Table S4. Risk (hazard ratios) of gastrointestinal cancers associated with indirect bilirubin levels in the UK Biobank

Table S5. Risk (hazard ratios) of gastrointestinal cancers associated with circulating bilirubin levels adjusted by liver enzymes in the UK Biobank

Table S1. The association between circulation total bilirubin levels and gastrointestinal cancers risk across strata of potential effect modifiers in the UK Biobank

Cancers	Groups	P-heterogeneity	HR (95% CI)	P
**Colorectal cancer (CRC)**				
Sex		0.48		
Smoking status		0.15		
Age		0.32		
	age< 50		0.83 (0.63-1.10)	0.19
	age>= 50		0.96 (0.89-1.03)	0.25
BMI_med*		0.29		
**Colon cancer**				
Sex		0.30		
Smoking status		0.30		
BMI_med*		0.36		
**Rectal cancer**				
Sex		>0.9		
Smoking status		0.26		
BMI_med*		0.72		
**Esophageal adenocarcinoma (EAC)**				
Sex		0.55		
Smoking status		0.26		
BMI_med*		0.77		
**Esophageal squamous-cell carcinoma (ESCC)**				
Sex		0.80		
Smoking status		0.01		
	Never		0.49 (0.22-1.09)	0.1
	Previous		0.97 (0.44-2.18)	>0.9
	Current		7.38 (0.59-92.56)	0.1
BMI_med*		N/A		
**Stomach cardia cancer**				
Sex		>0.9		
Smoking status		0.31		
BMI_med		>0.9		
**Stomach non-cardia cancer**				
Sex		>0.9		
Smoking status		N/A		
BMI_med*		0.61		
**Oral cancer**				
Sex		>0.9		
Smoking status		0.27		
BMI_med*		0.53		
**Pancreatic cancer**				
Sex		0.05		
	Men		0.83 (0.65-1.04)	0.1

	Women		1.11 (0.89-1.38)	0.4
Smoking status		0.008		
	Never		0.80 (0.63-1.00)	0.05
	Previous		1.16 (0.90-1.48)	0.3
	Current		1.09 (0.64-1.85)	0.8
BMI_med*		0.57		
**Hepatocellular carcinoma (HCC)**				
Sex		0.07		
Smoking status		0.25		
BMI_med*		0.67		
**Intrahepatic bile duct cancer (IBD)**				
Sex		0.56		
Smoking status		0.73		
BMI_med*		0.0004		
	<26.74		0.81 (0.43-1.51)	0.5
	>=26.74		4.36 (1.89-10.05)	0.001

HR: hazard ratio, CI: confidence interval, N/A: Not Available or Not Assessed.
Adjusted HR per 1-SD increment in circulating log-total bilirubin levels.
*Median values: BMI = 26.74
For these subgroup analyses, we used a more stringent P-value<0.002 to correct for multiple testing (0.05 divided by the number of tests [n=24]).
Formal test for heterogeneity by age groups were not possible due to low number of events in the age<50 except for CRC.

Table S2. Risk (hazard ratios) of gastrointestinal cancers associated with circulating bilirubin levels corrected for regression dilution in the UK Biobank

Cancers	HR (95%CI)*	HR (95%CI)**	P
**Colorectal cancer (CRC)**	0.95 (0.88-1.02)	0.93 (0.84-1.03)	0.15
**Colon cancer**	0.95 (0.87-1.04)	0.93 (0.83-1.05)	0.26
**Rectal cancer**	0.94 (0.83-1.07)	0.92 (0.77-1.10)	0.36
**Esophageal adenocarcinoma (EAC)**	0.72 (0.56-0.92)	0.63 (0.44-0.89)	0.01
**Esophageal squamous-cell carcinoma (ESCC)**	0.74 (0.44-1.24)	0.66 (0.32-1.35)	0.25
**Stomach cardia cancer**	1.33 (0.84-2.11)	1.48 (0.78-2.82)	0.23
**Stomach non-cardia cancer**	0.88 (0.41-1.91)	0.84 (0.29-2.45)	0.75
**Oral cancer**	0.93 (0.77-1.13)	0.90 (0.69-1.18)	0.46
**Pancreatic cancer**	0.96 (0.82-1.13)	0.95 (0.76-1.18)	0.62
**Hepatocellular carcinoma (HCC)**	2.07 (1.15-3.73)	2.75 (1.21-6.22)	0.02
**Intrahepatic bile duct (IBD) cancer**	1.67 (1.07-2.62)	2.04 (1.09-3.81)	0.03

HR: hazard ratio, CI: confidence interval.
Regression dilution ratio (RDR) value = 0.723 calculated as (mean follow-up total bilirubin in 5th Quintile - mean follow-up total bilirubin in 1st Quintile) /(mean baseline total bilirubin in 5th Quintile - mean baseline total bilirubin in 1st Quintile)
* Adjusted HR per 1-SD increment in circulating log-total bilirubin levels
**Adjusted HR per 1-SD increment in circulating log-total bilirubin levels corrected for regression dilution

Table S3. Risk (hazard ratios) of gastrointestinal cancers associated with circulating direct bilirubin levels in the UK Biobank (N= 374,933)

Cancers	Both sexes	Men	Women
**Colorectal cancer (CRC)**			
N cases	2,570	1,611	959
HR (95%CI)	0.95 (0.88-1.03)	0.95 (0.87-1.05)	0.97 (0.85-1.11)
P	0.23	0.31	0.68
**Colon cancer**			
N cases	1,690	987	703
HR (95%CI)	0.96 (0.87-1.06)	0.96 (0.85-1.09)	0.99 (0.84-1.16)
P	0.43	0.53	0.88
**Rectal cancer**			
N cases	880	624	256
HR (95%CI)	0.92 (0.80-1.05)	0.92 (0.79-1.08)	0.85 (0.61-1.18)
P	0.21	0.30	0.33
**Esophageal adenocarcinoma (EAC)**			
N cases	302	265	37
HR (95%CI)	0.83 (0.65-1.04)	0.88 (0.68-1.13)	N/A
P	0.11	0.32	N/A
**Esophageal squamous-cell carcinoma (ESCC)**			
N cases	102	56	46
HR (95%CI)	1.12 (0.67-1.88)	1.39 (0.50-3.84)	0.82 (0.29-2.34)
P	0.67	0.53	0.72
**Stomach cardia cancer**			
N cases	130	108	22
HR (95%CI)*	0.96 (0.59-1.54)	1.11 (0.62-1.97)	N/A
P	0.85	0.73	N/A
**Stomach non-cardia cancer**			
N cases	78	53	25
HR (95%CI)	0.81 (0.30-2.24)	N/A	N/A
P	0.69	N/A	N/A
**Oral cancer**			
N cases	457	319	138
HR (95%CI)	1.04 (0.85-1.28)	1.05 (0.80-1.37)	1.15 (0.80-1.63)
P	0.70	0.72	0.45
**Pancreatic cancer**			
N cases	491	284	207
HR (95%CI)	0.92 (0.77-1.10)	0.79 (0.61-1.01)	1.12 (0.86-1.46)
P	0.34	0.06	0.41
**Hepatocellular carcinoma (HCC)**			
N cases	129	106	23
HR (95%CI)	2.73 (1.15-6.49)	2.65 (1.07-6.54)	N/A
P	0.02	0.04	N/A
**Intrahepatic bile duct (IBD) cancer**			
N cases	106	53	53
HR (95%CI)	1.77 (1.11-2.82)	9.03 (0.75-109.38)	1.41 (0.76-2.63)
P	0.02	0.08	0.28

HR: hazard ratio, CI: confidence interval, N/A: Not Available or Not Assessed.
Hazard Ratio (HR) per 1-SD increment in circulating log-total bilirubin levels.

Table S4. Risk (hazard ratios) of gastrointestinal cancers associated with indirect bilirubin levels in the UK Biobank (N= 374,933)

Cancers	Both sexes	Men	Women
**Colorectal cancer (CRC)**			
N cases	2,570	1,611	959
HR (95%CI)	0.97 (0.90-1.05)	0.93 (0.85-1.03)	1.06 (0.93-1.21)
P	0.42	0. 15	0.39
**Colon cancer**			
N cases	1,690	987	703
HR (95%CI)	0.98 (0.89-1.07)	0.93 (0.83-1.05)	1.08 (0.92-1.26)
P	0.66	0.25	0.33
**Rectal cancer**			
N cases	880	624	256
HR (95%CI)	0.93 (0.81-1.06)	0.91 (0.77-1.07)	0.93 (0.69-1.28)
P	0.28	0.23	0.69
**Esophageal adenocarcinoma (EAC)**			
N cases	302	265	37
HR (95%CI)	0.73 (0.56-0.95)	0.74 (0.56-0.99)	N/A
P	0.02	0.04	N/A
**Esophageal squamous-cell carcinoma (ESCC)**			
N cases	102	56	46
HR (95%CI)	0.60 (0.31-1.16)	0.31 (0.08-1.25)	0.65 (0.25-1.70)
P	0.13	0.10	0.38
**Stomach cardia cancer**			
N cases	130	108	22
HR (95%CI)	1.21 (0.76-1.92)	1.53 (0.83-2.83)	N/A
P	0.43	0.17	N/A
**Stomach non-cardia cancer**			
N cases	78	53	25
HR (95%CI)	0.68 (0.24-1.91)	N/A	N/A
P	0.46	N/A	N/A
**Oral cancer**			
N cases	457	319	138
HR (95%CI)	0.91 (0.73-1.14)	0.92 (0.70-1.21)	0. 97 (0.64-1.47)
P	0.41	0.57	0.88
**Pancreatic cancer**			
N cases	491	284	207
HR (95%CI)	0.89 (0.74-1.08)	0.81 (0.62-1.05)	1.03 (0.78-1.36)
P	0.24	0.11	0.83
**Hepatocellular carcinoma (HCC)**			
N cases	129	106	23
HR (95%CI)	1.46 (0.81-2.63)	1.09 (0.52-2.26)	N/A
P	0.21	0.82	N/A
**Intrahepatic bile duct (IBD) cancer**			
N cases	106	53	53
HR (95%CI)	1.41 (0.87-2.26)	4.48 (0.78-25.80)	1.11 (0.56-2.17)
P	0.16	0.09	0.77

HR: hazard ratio, CI: confidence interval, N/A: Not Available or Not Assessed.
Adjusted HR per 1-SD increment in circulating log-total bilirubin levels.
To calculate indirect bilirubin, total bilirubin levels are subtracted from the direct bilirubin levels.

Table S5. Risk (hazard ratios) of gastrointestinal cancers associated with circulating bilirubin levels adjusted by liver enzymes in the UK Biobank (N= 439,851)

Cancers	HR (95%CI)*	HR (95%CI)**	P
**Colorectal cancer (CRC)**	0.95 (0.88-1.02)	0.95 (0.86-1.02)	0.16
**Colon cancer**	0.95 (0.87-1.04)	0.95 (0.87-1.04)	0.28
**Rectal cancer**	0.94 (0.83-1.07)	0.94 (0.83-1.07)	0.36
**Esophageal adenocarcinoma (EAC)**	0.72 (0.56-0.92)	0.72 (0.55-0.93)	0.01
**Esophageal squamous-cell carcinoma (ESCC)**	0.74 (0.44-1.24)	0.74 (0.44-1.24)	0.25
**Stomach cardia cancer**	1.33 (0.84-2.11)	1.33 (0.84-2.11)	0.23
**Stomach non-cardia cancer**	0.88 (0.41-1.91)	0.88 (0.41-1.91)	0.75
**Oral cancer**	0.93 (0.77-1.13)	0.92 (0.76-1.12)	0.40
**Pancreatic cancer**	0.96 (0.82-1.13)	0.95 (0.82-1.13)	0.66
**Hepatocellular carcinoma (HCC)**	2.07 (1.15-3.73)	2.07 (1.15-3.73)	0.02
**Intrahepatic bile duct (IBD) cancer**	1.67 (1.07-2.62)	1.67 (1.07-2.62)	0.03

HR: hazard ratio, CI: confidence interval.

* Adjusted HR per 1-SD increment in circulating log-total bilirubin levels

**Adjusted HR per 1-SD increment in circulating log-total bilirubin levels after exclusion of participants with liver enzymes beyond 90th percentile (including alanine transaminase (ALT), aspartate transaminase (AST), alkaline phosphatase (ALP), and gamma-glutamyl transpeptidase (GGT).

N Colorectal cancer: 2,998, N Colon cancer: 1,987, N Rectal cancer: 1,011, N Esophageal adenocarcinoma: 336, N Esophageal squamous-cell carcinoma: 124, N Stomach cardia cancer: 139, N Stomach non-cardia cancer: 92, N Oral cancer: 523, N Pancreatic cancer: 556, N Hepatocellular carcinoma: 135, N Intrahepatic bile duct cancer: 120.
